# ManCAD100: 100 Years of Audiology and Deaf Education at Manchester

**DOI:** 10.1177/2331216519886239

**Published:** 2019-11-27

**Authors:** Christopher J. Plack, Helen Chilton, Kevin J. Munro

**Affiliations:** 1 Manchester Centre for Audiology and Deafness, The University of Manchester, Manchester Academic Health Science Centre, UK; 2 NIHR Manchester Biomedical Research Centre, Central Manchester University Hospitals NHS Foundation Trust, Manchester Academic Health Science Centre, UK

**Keywords:** audiology, deaf education, Manchester, Ewing

## Abstract

In 2019, the Manchester Centre for Audiology and Deafness celebrates its 100th anniversary. To mark the centenary, this special issue is a collection of papers that showcases current research in Manchester Centre for Audiology and Deafness. The Editorial provides a brief history and description of the Centre and an overview of the special issue.

## ManCAD Past and Present

The Department of Education for the Deaf was founded at The University of Manchester in 1919. It was the first university department of deaf education in the world. The department was funded by a donation from the estate of Ellis Llwyd Jones, who was born deaf, by his father, businessman Sir James Jones. Irene Goldsack (later Ewing) was the first Ellis Llwyd Jones lecturer. Irene Ewing and her husband Alexander were responsible for the fundamental concepts of pediatric audiology. They also influenced professionals throughout the world on issues pertaining to the identification and management of childhood deafness. In 1947, Irene Ewing was awarded the Order of the British Empire for her services to audiology and deaf education. In 1958, Alexander Ewing was knighted for his services to audiology and deaf education.

The department has made a number of seminal contributions for patient benefit, particularly with respect to pediatric diagnostics, auditory devices, and deaf education. In 1928, the department imported an electronic audiometer from the United States and conducted groundbreaking audiometry research, showing in particular that deaf children usually have residual low-frequency hearing that can be enhanced by hearing aids. Physicist Tom Littler from the department was a pioneer in the design of electronic hearing aids. In 1933, he developed a group hearing aid for use in classrooms and then helped to develop individual aids (under the *Medresco* brand) that were issued free of charge by the U.K. National Health Service (NHS) in its foundation year, 1948. In 1944, the Ewings developed the *Distraction Test* (sometimes called the *Ewing Test*), which reduced the age of diagnosis of hearing loss from 2 to 3 years to 6 months. The Distraction Test was used around the world until the 1990s. The Ewings practiced and advocated for a child-centered focus when teaching deaf children. This was seen to be unusual, but innovative, as their methods were far removed from the standard practice of speech drills and rote learning. In 1944, Irene Ewing demonstrated the linguistic and developmental advantages displayed by children whose parents had engaged in their early support clinics. The foundations of early, meaningful interaction are held central to all approaches in the field to this day.

In 1964, Ian Taylor (who replaced the recently retired Alexander Ewing) published *The Neurological Mechanisms of Hearing and Speech in Children* (Taylor, 1964), which was one of the first reports of what is now known as “auditory neuropathy spectrum disorder.” Around this time, there was an expansion of interests to include speech pathology and audiological medicine, with studies on epidemiology, etiology, and auditory genetics. In the 2000s, the department was instrumental in the development and delivery of universal newborn hearing screening, based on measurements of otoacoustic emissions and auditory brainstem responses, which replaced the Distraction Test. Continuing its involvement in auditory devices, the department led the modernization of children’s hearing-aid services in the NHS and recently established the Hearing Device Research Centre. From 1988 until 2013, the department also hosted the Manchester Cochlear Implant Centre, the largest auditory implant center in the United Kingdom, providing clinical services to adults and children. The Centre performed the first auditory brainstem implant for a child. In 2008, the Centre celebrated its 20th anniversary and 1,000th implant.

Today, the department, now known as the Manchester Centre for Audiology and Deafness (ManCAD), is stronger than ever, with 14 members of permanent academic staff. Our audiology research covers a wide range from the basic physiology of hearing and hearing impairment, noise-induced hearing loss, and hearing and cognition, to auditory devices, advanced diagnostics, and pediatric audiology. The translational work in deaf education continues to shape practice in the field and in recent years has focused on deaf children with complex needs, effective radio aid use, and the development of Theory of Mind skills for deaf children and young people. The impact of our research is underpinned by a close relationship with health and education service providers and with leading international manufacturers of hearing aids and cochlear implants. ManCAD is also a major provider of training for students of audiology and for teachers of the deaf.

Manchester has made a world-leading, and life-changing, contribution to audiology and deaf education in terms of education and training, research across the life span, service development and practice. To mark the ManCAD centenary, Laura Dawes, medical historian, author, and broadcaster, has updated and revised her history of audiology and deaf education at The University of Manchester (Dawes, 2019).

## Overview of the Special Issue

The special issue showcases a range of current audiology research in ManCAD. Advanced diagnostics has long been a core component of our work at Manchester, and the issue includes several papers on this theme. The paper by Moore et al. (2019) describes a new speech-in-noise assessment tool, FreeHear, which shows promise as a rapid and reliable measure of listening ability. Guest et al. (2019) examine the effects on acoustic reflex thresholds of interstimulus interval, a parameter that has received little attention to date. The review by McDermott et al. (2019) exemplifies an important new direction for the group in genomics research, with huge potential for pediatric diagnostics.

Similarly, our historical research strength in auditory devices continues to the present day. Two papers examine the use of electrophysiological responses in the evaluation of hearing aids. The study by BinKhamis, Elia Forte, Reichenbach, O’Driscoll, and Kluk (2019) suggests that although the speech auditory brainstem response may not be a good predictor of listening ability, it may have some value as an objective measure of aided speech detection. Stone, Visram, Harte, and Munro (2019) examine a set of speech-like stimuli that may have clinical utility for the assessment of hearing-aid performance via cortical-evoked potentials. With direct relevance to patients, Almufarrij, Munro, Dawes, Stone, and Dillon (2019) provide an assessment of direct-to-consumer hearing devices, which can be obtained without consulting a hearing health professional. The challenge for manufacturers is to develop low-cost products with cosmetic appeal and appropriate electroacoustic characteristics.

The city of Manchester has a somewhat notorious reputation as a source of both occupational (cotton mills) and recreational (Joy Division, The Smiths, The Stone Roses, Oasis, Manchester City and Manchester United soccer stadiums) noise exposure. Perhaps appropriately, the effect of noise exposure on hearing health is a strong current interest in the group. The paper by Prendergast et al. (2019) is a continuation of our research in noise-induced cochlear synaptopathy, although we still struggle to find good evidence for the condition in humans. The UK Biobank, containing health data for half a million people, is a hugely powerful resource for health-care research, and we are pioneers in its use for determining the factors associated with hearing deficits. The study by Couth, Mazlan, Moore, Munro, and Dawes (2019) uses Biobank data to examine the levels of hearing difficulties and tinnitus in high-risk industrial settings (e.g., music, construction, and agricultural), as well as investigating other demographic, health, and lifestyle risk factors.

The papers illustrate the wide range of different approaches used in our research, including genetic testing, audiometry, electrophysiology, psychophysics, speech testing, subjective quality assessments, questionnaire-based techniques, and the use of big data. With emerging cross-disciplinary projects in cancer treatments, biomarkers, genomics, health informatics and data science, artificial intelligence, health psychology, engineering, and advanced materials, we continue to explore exciting new directions in audiology research. The expectation is that our next 100 years will be even more transformative than the first.
